# Intraoperative allogeneic transfusion is associated with postoperative delirium in older patients after total knee and hip arthroplasty

**DOI:** 10.3389/fsurg.2022.1048197

**Published:** 2023-01-05

**Authors:** Chun-lei OuYang, Xin-yu Hao, Yao Yu, Jing-sheng Lou, Jiang-bei Cao, Ying-qun Yu, Wei-dong Mi

**Affiliations:** ^1^ Department of Anesthesiology, The First Medical Center of PLA General Hospital, Beijing, China; ^2^ Medical School of Chinese PLA, Beijing, China; ^3^ Department of Anesthesiology, The Fifth Medical Center of PLA General Hospital, Beijing, China

**Keywords:** intraoperative allogeneic transfusion, delirium, anesthesiology, total hip arthroplasty, total knee arthroplasty

## Abstract

**Objective:**

To determine whether intraoperative transfusion of allogeneic or autologous blood is associated with an increased incidence of postoperative delirium (POD) after total knee arthroplasty (TKA) and total hip arthroplasty (THA).

**Methods:**

The medical records of 1,143 older (≥65 years old) patients who received an intraoperative blood transfusion while undergoing total knee or hip arthroplasty at the First Medical Center of Chinese PLA General Hospital from 2014 to 2019 were reviewed; of these patients, 742 (64.92%) received allogeneic blood, while 401 (35.08%) received autologous blood. Patients who received autologous transfusion were paired with those received allogeneic transfusion using 1:1 propensity score matching method. The primary outcome was POD. The secondary outcomes were postoperative complications, including heart failure, deep vein thrombosis, myocardial infarction, stroke, and lung infection. Multivariable nominal logistic regression was used to identify any independent associations between intraoperative blood transfusions and POD, and secondary postoperative complications, respectively.

**Results:**

Postoperative delirium occurred in 6.6% (49/742) of patients who had received an allogeneic blood transfusion and in 2.0% (8/401) of patients who had received an autologous blood transfusion. It is noteworthy that the multivariable logistic regression demonstrated a significant association between intraoperative allogeneic blood transfusion and POD (odds ratio [OR]: 4.11; 95% confidence interval [CI]: 1.95–9.77; *p* < 0.001). After PSM, Allogeneic transfusion was also the strongest predictor for POD (OR: 4.43; 95% CI: 2.09–10.58; *p* < 0.001).

**Conclusions:**

In the patients who had received THA or TKA, intraoperative allogeneic blood transfusions were associated with an increased risk of POD.

## Introduction

Postoperative delirium (POD), defined as an acute state of confusion characterized by fluctuating consciousness and inattention (1), has attracted increasing global interest and prompted extensive research on its prevention and management (2). With a reported incidence of 2%–30%, POD is associated with an increased risk of mortality and a prolonged hospital stay in addition to reduced functioning, lower quality of life, and exorbitant hospital costs, resulting in significant implications for both patients and healthcare systems (3, 4). This condition requires considerable attention from both clinicians and patients.

Understanding the etiology and identifying the risk factors for POD could inform interventions to improve the outcomes for elderly patients undergoing surgery. Many risk factors associated with POD have been identified. Unmodifiable factors, such as age and comorbidity burdens, are commonly studied risk factors for POD. However, the role of modifiable factors, such as commonly used perioperative medications and intraoperative blood transfusions, remains uncertain (5–8). Several studies have shown the risk factors for POD may be related to a variety of blood flow variables, but it has not been shown whether allogeneic or autologous blood has more influence on POD (9, 10). Allogeneic blood transfusion can correct intraoperative anemia, while increases the risk of bacterial contamination, transfusion transmitted diseases, and transfusion-related immunological diseases (11, 12). Intraoperative autologous blood transfusion has been gradually attached importance and popularized in recent years due to its advantages of convenience and having no influence on coagulation function (13, 14).

Usually, total joint arthroplasty (TJA), which is an effective procedure in the treatment of various joint pathologies, is associated with substantial blood loss and a requirement for a blood transfusion (15). The reported incidence of transfusion varies from 3.5% to 18.5% for TKA and from 5.4% to 26.2% for THA (16). Therefore, we hypothesized that intraoperative blood transfusion, a known trigger and amplifier of inflammation, is an independent risk factor for early POD in older patients undergoing surgery. Owing to the surgical complexity of TJA, patients undergoing TJA have an especially high risk of developing POD (17–20).

However, it remains relatively unknown whether the type of intraoperative blood transfusion used increases the risk of POD after TJA. In this retrospective study, we aimed to determine whether an intraoperative allogeneic blood transfusion increases the incidence of POD in patients with TJA compared with an intraoperative autologous blood transfusion.

## Methods

### Study design and data source

The medical records of 1,143 adult (≥65 years of age) patients undergoing primary TJA with normal preoperative cognitive function at the Chinese People's Liberation Army General Hospital between January 2014 and August 2019 were reviewed retrospectively. All the data were obtained from the medical and nursing records in the hospital's perioperative database. This study was approved by the Institutional Review Board (reference number: S2021-342-01). The reliability and accuracy of the data extracted from the medical records were ensured using trained surgical clinical reviewers, who adhered to strict data definitions.

### Inclusion and exclusion criteria

The inclusion criteria were: (1) age ≥65 years, (2) patients undergoing their first TJA with normal preoperative cognitive function, (3) patients suffering from hip or knee osteoarthritis and femoral head necrosis, (4) only the first procedure was included for patients who underwent staged TJAs for both lower limbs, and (5) patients had not received a preoperative blood transfusion. The events of the perioperative transfusion were recorded. The exclusion criteria were patients with tibial fractures, hemorrhage, or coagulation disorders; a previous history of thrombosis or hemorrhage; liver or kidney function abnormalities; severe lesions of the mental and nervous system; a cardiac pacemaker, or an *in vivo* stent.

### Baseline characteristics and comorbidities

The baseline characteristics evaluated included patients' age, gender, American Society of Anesthesiologists (ASA) physical status classification, and body mass index. One group comprised patients who were classified as ASA I or ASA II, while the other group comprised patients who were ASA III or ASA IV. Classifications were combined because the patients classified as ASA I (*n* = 5) and ASA IV (*n* = 7) were too small in number to be analyzed as separate groups. Intraoperative blood loss was calculated as the volume of blood in a suction canister plus the estimated amount of blood contained in surgical sponges. The intraoperative factors included the duration of the surgery and the blood loss. The preoperative laboratory values included preoperative hemoglobin (Hgb), white blood cells (WBC), platelets, and hematocrit (HCT).

Comorbidities included smoking history, diabetes, congestive heart failure, prior myocardial infarction (MI), hypertension (HTN), chronic obstructive pulmonary disorder (COPD), and stroke. Hip replacement was performed by the same surgical team, and a cemented double prosthesis was used *via* a posterior lateral approach in all cases. According to “Beijing”s Guidelines for Clinical Blood Transfusion”, the intraoperative allogeneic blood transfusion trigger was an Hgb level of less than 10.0 g/dl (21).

### Outcomes and definitions

Postoperative delirium was the primary outcome investigated. The secondary outcomes were lung infection, deep vein thrombosis (DVT), MI, stroke, and heart failure. Delirium was captured through descriptive words documented in the medical chart, including “mental status change,” “confusion,” “disorientation,” “agitation,” “delirium,” “inappropriate behavior,” “inattention,” “hallucinations,” and “combative behavior” (22). The occurrence of delirium was defined as the patient meeting any of the above criteria on any of the postoperative day assessments conducted postoperatively during the daytime (22). The incidence of POD in patients after TKA/THA surgery was first captured by extracting the above descriptive words from the medical charts using database program. The symptoms of delirium were further confirmed by a neurologist to ensure the data quality. Lung infection was defined as antibiotic treatment administered for suspected pneumonia plus at least one of the following: new/changed sputum, new/changed radiographic opacities, temperature >38.3 °C, or leukocyte count >12,000/mm^3^ (23).

### Statistical analysis

Continuous variables were presented as median with interquartile range (IQR) and the differences between groups were identified with the Mann–Whitney *U* test because of non-normal distribution. Categorical variables were presented as numbers and percentages, and compared using the chi-square test or Fisher's exact test as appropriate. The patients were grouped according to whether they had received an autologous or allogeneic blood transfusion during operation, the differences of patients baseline characteristics and outcomes were compared between groups.

To evaluate the correlation between the type of transfusion and outcomes among older patients undergoing total joint arthroplasty, propensity score matching was carried out in this study using a nearest neighbor matching method with a 0.2 caliper in a 1:1 ratio. The balance between the matched groups was assessed by calculating the standardized mean difference (SMD). The following variables were used to calculate the propensity score: age, ASA class, Hb, WBC, HCT, duration of operation. The independent association between the transfusion type and the outcomes with complications (primary and secondary) was examined using logistic regression model adjusting for confounding variables with *p* value less than 0.05 in univariate analysis. The impact of transfusion type on outcome events was quantified by the odds ratio (OR) and 95% confidence interval (CI).

All statistical analyses were performed using R statistical software (R version 4.0.5). For all tests, a two-tailed *p* value of < 0.05 was considered statistically significant.

## Results

### Patient demographic and baseline characteristics

**Figure 1:**
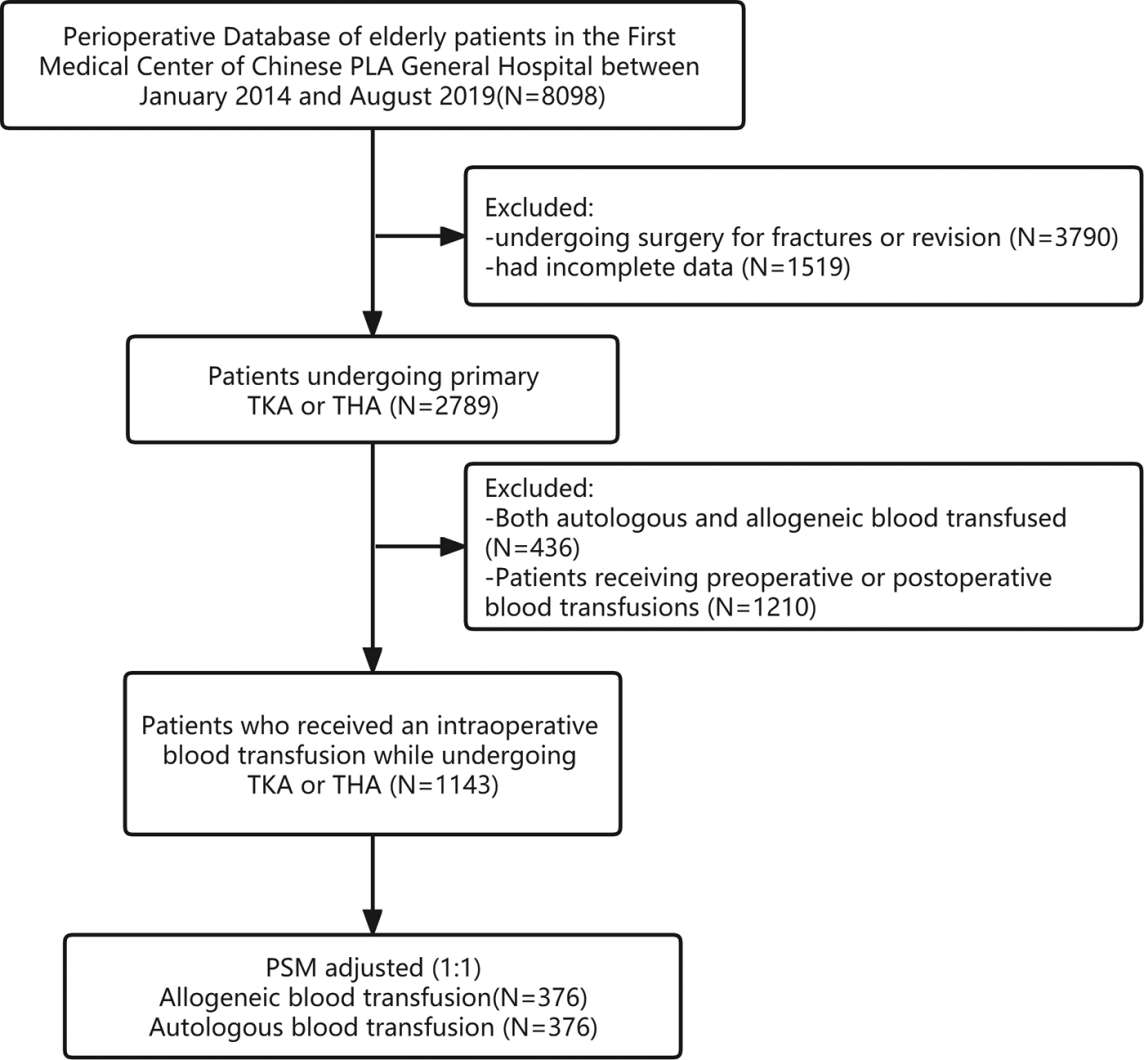
Flowchart for inclusion and exclusion of study subjects. THA, total hip arthroplasty; TKA, total knee arthroplasty.

The medical records of 2,789 patients over 65 years old with TKA or THA in the Department of Orthopedics of the Chinese People's Liberation Army General Hospital from January 2014 to August 2019 were retrospectively analyzed. The flowchart (see Figure 1) shows that after a series of exclusions, a total of 1,143 older patients who had undergone TKA or THA were included in this study (autologous blood transfusion: *n* = 401; allogeneic blood transfusion: *n* = 742). In the autologous group, TKA surgery accounted for 82.0%, and THA surgery accounted for 18.0%. In the allogeneic group, TKA surgery accounted for 76.7%, and THA surgery accounted for 23.3%.

As shown in Table 1, patients who received allogeneic blood were more likely to have coronary heart disease and higher ASA physical status. The levels of preoperative hemoglobin and hematocrit were significantly lower among patients with allogeneic blood transfusion. Operations on both sides were more likely to be performed on patients with allogeneic blood transfusion. They have longer operation time and more blood loss. There were no significant differences in sex, history of drinking, smoking, and preoperative comorbidities (diabetes, hypertension, COPD, cerebro- and peripheral vascular diseases, and stroke).

**Table 1:** Comparisons between patients with allogeneic and autologous transfusion before and after PSM.

Characteristic	Before PSM				After PSM (1:1)			
	Autologous (*n* = 401)	Allogeneic (*n* = 742)	*P*	SMD	Autologous (*n* = 376)	Allogeneic (*n* = 376)	*P*	SMD
**Demographic Information**								
Age, median (IQR), years	68 (66–71)	69 (67–74)	<0.001	0.374	68 (66–71)	68 (66–72)	0.984	0.060
ASA physical status, *n* (%)								
I–II	375 (93.5)	647 (87.2)	0.010	0.219	350 (93.1)	355 (94.4)	0.674	0.090
III–IV	26 (6.5)	95 (12.8)			26 (6.9)	21 (5.6)		
Smoking, *n* (%)	74 (18.5)	117 (15.8)	0.281	0.071	67 (17.8)	58 (15.4)	0.433	0.064
Drinking, *n* (%)	74 (18.5)	112 (15.1)	0.166	0.090	67 (17.8)	56 (14.9)	0.324	0.079
BMI, median (IQR), kg/m^2^,	26.6 (24.5–29.0)	26.4 (24.0–28.9)	0.159	0.062	26.7 (24.6–29.0)	26.4 (24.2–28.9)	0.357	0.043
Female, *n* (%)	293 (73.1)	547 (73.7)	0.866	0.015	281 (74.7)	269 (71.5)	0.365	0.072
**Comorbidities**								
COPD, *n* (%)	4 (1.0)	16 (2.2)	0.234	0.093	4 (1.1)	9 (2.4)	0.263	0.102
Diabetes, *n* (%)	75 (18.7)	151 (20.4)	0.556	0.042	67 (17.8)	67 (17.8)	1.000	0.001
Hypertension, *n* (%)	212 (52.9)	419 (56.5)	0.269	0.072	198 (52.7)	224 (59.6)	0.066	0.140
Heart failure, *n* (%)	5 (1.2)	10 (1.3)	1.000	0.009	5 (1.3)	7 (1.9)	0.771	0.042
Myocardial infarction, *n* (%)	3 (0.7)	22 (3.0)	0.026	0.165	2 (0.5)	13 (3.5)	0.009	0.210
Coronary heart disease, *n* (%)	21 (5.2)	77 (10.4)	0.004	0.192	20 (5.3)	48 (12.8)	0.001	0.262
Peripheral vascular diseases, *n* (%)	44 (11.0)	107 (14.4)	0.121	0.104	43 (11.4)	55 (14.6)	0.233	0.095
Cerebrovascular diseases, *n* (%)	5 (1.2)	4 (0.5)	0.346	0.075	5 (1.3)	3 (0.8)	0.722	0.052
Stroke, *n* (%)	29 (7.2)	80 (10.8)	0.065	0.124	23 (6.1)	31 (8.2)	0.323	0.101
**Preoperative laboratory tests**								
Hemoglobin, median (IQR), g/l	131.0 (122.0–140.0)	128.0 (117.0–137.0)	<0.001	0.311	129.5 (122.0–138.0)	131.0 (122.0–141.0)	0.410	0.009
WBC count, median (IQR), 10^9^/l	5.89 (4.96–7.08)	5.87 (5.02–7.07)	0.066	0.052	5.82 (4.96–7.00)	5.78 (4.94–6.93)	0.559	0.036
Hematocrit, median (IQR), %	39 (37–42)	38 (35–41)	<0.001	0.350	39 (37–41)	39 (37–42)	0.506	0.021
Platelets, median (IQR), 10^9^/l	227 (188–259)	223 (190–264)	0.654	0.090	227 (188–258)	214 (180–261)	0.220	0.023
**Intraoperative information**								
Duration of operation, median (IQR), mins	120 (100–173)	145 (110–215)	<0.001	0.370	125 (105–180)	125 (100–178)	0.764	0.016
Surgical site, *n* (%)								
On one side	332 (82.8)	509 (68.6)	<0.001	0.336	307 (81.6)	279 (74.2)	0.018	0.180
On both sides	69 (17.2)	233 (31.4)			69 (18.4)	97 (25.8)		
Blood loss, median (IQR), ml	200 (200–400)	300 (200–500)	<0.001	0.347	200 (200–400)	300 (200–400)	0.04	0.195
Anesthesia method, *n* (%)								
Combined general anesthesia	209 (52.1)	359 (48.4)	0.098	0.154	199 (52.9)	190 (50.5)	0.052	0.204
General anesthesia alone	150 (37.4)	326 (43.9)			136 (36.2)	163 (43.4)		
Nerve blocks	22 (5.5)	34 (4.6)			22 (5.9)	12 (3.2)		
Epidural anesthesia	20 (5.0)	23 (3.1)			19 (5.1)	11 (2.9)		
**Postoperative outcome**								
POD, *n* (%)	8 (2.0)	49 (6.6)	0.001	0.229	8 (2.1)	37 (9.8)	<0.001	0.330
Deep vein thrombosis, *n* (%)	3 (0.7)	7 (0.9)	0.996	0.021	3 (0.8)	4 (1.1)	1.000	0.028
Secondary surgery, *n* (%)	1 (0.2)	2 (0.3)	1.000	0.004	1 (0.3)	1 (0.3)	1.000	<0.001
Second hospitalization, *n* (%)	1 (0.2)	9 (1.2)	0.181	0.113	1 (0.3)	5 (1.3)	0.219	0.120
Lung infection, *n* (%)	2 (0.5)	21 (2.8)	0.014	0.183	2 (0.5)	10 (2.7)	0.042	0.170

PSM, propensity score matching; SMD, standardized mean difference; IQR, interquartile range; ASA, American society of anesthesiologists; BMI, body mass index; COPD, chronic obstructive pulmonary disease; WBC, white blood cell; POD, postoperative delirium.

### POD and secondary outcomes

There was a significant higher incidence of POD (autologous group: 2.0% vs. allogeneic group: 6.6%; *p* < 0.001) and lung infection (autologous group: 0.5% vs. allogeneic group: 2.8%; *p* = 0.014) among patients who had received allogeneic blood transfusion (see Table 1). The incidence of other postoperative complications (deep vein thrombosis, reoperation, and rehospitalization) were not statistically significant between groups (see Table 1).

### Relationship between blood transfusion type and outcomes

Logistic regression model was used to examine the differences of postoperative outcomes between the two groups. In the unmatched cohort, allogeneic blood transfusion was associated with increased likelihood of developing POD (OR: 4.11; 95% CI: 1.95–9.77; *p* < 0.001) in multivariate logistic analysis after controlling for confounders, including age, sex, body mass index, ASA physical status, smoking history, diabetes, preoperative levels of hemoglobin and white blood cells, duration of operation, and blood loss. The impact of blood transfusion type on other postoperative complications was estimated, and the results showed that allogeneic transfusion was not associated with lung infection (OR: 4.28; 95% CI: 1.08–28.45; *p* = 0.065) (see Table 2).

**Table 2:** Multivariate nominal logistic regression analysis of independent associations with and POD lung infection.

	OR	95% CI	*P*
**POD**			
Pre-matched model	4.11	1.95–9.77	<0.001
PSM model	4.43	2.09–10.58	<0.001
**Lung infection**			
Pre-matched model	4.28	1.08–28.45	0.065
PSM model	4.40	1.11–29.09	0.060

OR, odds ratio; CI, confidence interval; POD, postoperative delirium; PSM, propensity score matching.

**Figure 2:**
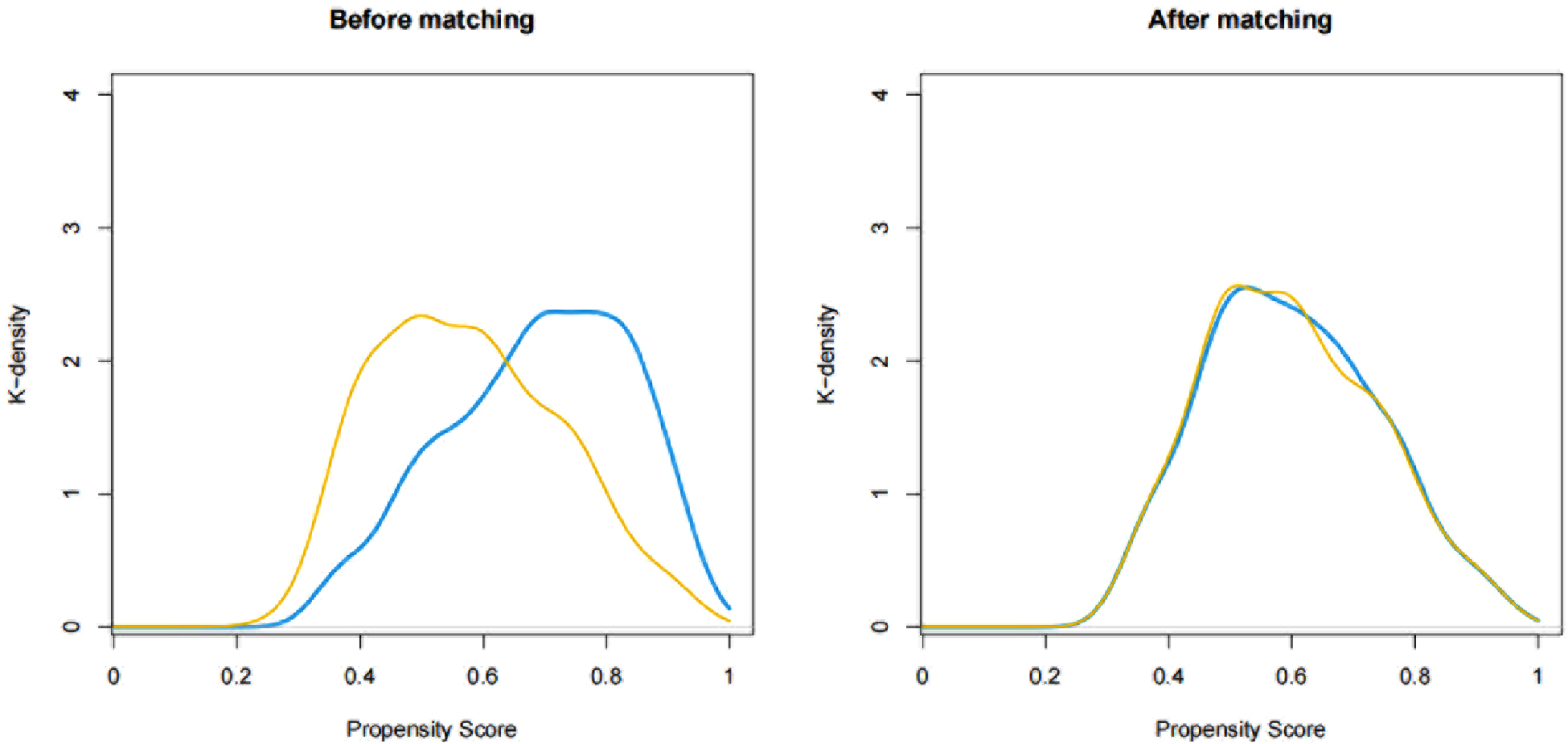
Distribution of propensity scores (**A**) before and (**B**) after matching.

In PSM, 376 patients who received allogeneic blood transfusion were matched with 376 patients who received autologous blood transfusion. The comparison between the matched groups were shown in Table 1. After propensity score matching, the patient baseline profiles were well balanced between the two groups with standardized mean differences less than 0.2 for nearly all variables (see Table 1 and Figure 2). Similar to the results in the pre-matched model, allogeneic blood transfusion was associated with increased risk of developing POD (OR: 4.43; 95% CI: 2.09–10.58; *p* < 0.001) (see Table 2) in multivariate logistic analysis after controlling for confounders, including sex, body mass index, smoking history, diabetes, and blood loss.

## Discussion

### Summary of key findings

This retrospective study evaluated the role of intraoperative allogeneic blood transfusion in the development of POD among older patients undergoing elective TJA. The results of this study showed that the incidence of POD in the allogeneic blood transfusion group (6.6%) was significantly higher than in the autologous blood transfusion group (2.0%). This result was similar to that of previous studies, which showed that between 2% and 15% of older people undergoing elective orthopedic surgery may develop POD (19, 24, 25).

### Association with previous studies

POD is a serious complication after major surgery in older patients, particularly those undergoing major arthroplasties (26). This condition is a serious event for patients, families, and healthcare workers, and is associated with negative outcomes including cognitive impairment, institutionalization, and death (27, 28). Older patients with TJA are at high risk for POD, of which incidence and risk factors vary across the literature. In a prospective study of 225 adults >70 years who underwent THA or TKA, Krenk et al. demonstrated that 18.6% patients developed POD, with risk factors including ASA classification, anesthesia method, postoperative pain, and opioid use (29). Analogously, in a retrospective study of 6,349 adult patients undergoing THA or TKA, Petersen et al. identified the significant risk factors for POD as age >75 years and length of hospital stay (30).

Many of the risk factors identified have been cited in previous reports and are not open to modification, e.g., advanced age, medical comorbidities, and a history of psychiatric disease. However, some risk factors, such as pre-existing narcotic dependence, alcoholism, and hyponatremia, are potentially modifiable. In addition to the surgical procedure, intraoperative blood transfusion may affect the incidence of POD and could be targeted to reduce it. In our study, POD occurred in 6.6% of patients, and there was a significant correlation between allogeneic blood transfusion and the risk of POD.

While the correlation between intraoperative blood transfusion and POD is relatively unknown in TJA, this risk stratification has been attempted in other surgical specialties. In a prospective study by Guo et al., who investigated the risk factors for POD after hip fracture surgery in 572 cases, it was found that intraoperative blood transfusion was an independent risk factor for the condition (31). In a retrospective study of 649 patients undergoing bilateral TKA, Soranoglou et al. found allogeneic blood transfusion to be associated with an increased risk of POD (OR: 3.10) (32). Similar to the aforementioned studies, the present study found the highest risk (OR: 4.43) of POD in TKA or THA to be associated with allogeneic blood transfusion only.

### Mechanisms of POD associated with blood transfusion

The pathophysiological relationship between allogeneic blood transfusion and POD may be attributed to the inflammatory response induced by blood transfusion in the patient. Although the pathogenesis of POD is not completely understood, many factors (e.g., hypoxia, cholinergic deficiency, or dopamine excess, dysregulation of the hypothalamic–pituitary–adrenal axis, and increased neuroinflammation) have been proposed. Among these, the inflammatory hypothesis emphasizes the role of cytokines in the development of POD, including TNF-α, IL-1, IL-6, and IL-8 (33). The neuroinflammatory hypothesis is based on the activation of neural inflammation within the brain due to peripheral, systemic, or sepsis-associated inflammation. The passage of peripheral inflammatory mediators into the central nervous system can result in the production of cytokines and inflammatory mediators within the central nervous system (34). Moreover, this neuroinflammatory response is associated with neurotransmitter disturbances, neuronal dysfunction, and the clinical manifestations of the POD syndrome. Although the specific mechanism is not yet clear, the immune response as a biological mediator between blood transfusion and POD may be a potential explanation for why allogeneic blood transfusion may pose a higher risk of the condition than autologous blood transfusion. One explanation for these unfavorable results may be that the long-term storage of red blood cells in allogeneic blood transfusions can lead to significant functional and structural changes, subsequently resulting in increased inflammation and oxidative damage (35).

Even recent guidelines advocating blood conservation continue to stress resource utilization and blood-borne viruses without much consideration of the elevation of postoperative morbidities, such as lung infections. Our research found that allogeneic blood transfusion is associated with a higher (but not statistically significant) risk of lung infection. Allogeneic blood is immunologically active, and various types of immunological and hypersensitivity reactions can occur following the transfusion of allogeneic blood. A common trait among these reactions is their involvement in the activation of immune system domains (36). Considering the immune suppression effect of blood components and metabolic products in the immune regulation of the body, the transfusion of leukocyte-removing filtered blood components should be selected to reduce the incidence of postoperative pulmonary infections in patients.

Patient blood management, which views the patient's own blood as a resource that should be conserved, is a patient-centered interdisciplinary approach that involves the timely application of evidence-based medical and surgical interventions designed to maintain the patient's own blood mass (37). It consists of three pillars: the optimization of red blood cell mass, the reduction of blood loss and bleeding, and the optimization of the patient's physiological tolerance toward anemia. The integration of these three pillars in the form of multimodal care bundles and strategies into perioperative pathways should improve care processes and patient outcomes (38).

Various process measures can be used to avoid blood transfusion, including the elevation of preoperative HCT in elective cases by the administration of iron and vitamin-B complex, and possibly, the selective use of erythropoietin (39). In addition, attention to intraoperative hemostasis, the establishment of protocols for transfusion and re-operation for bleeding, the use of antifibrinolytic drugs, and a tolerance for mild-to-moderate anemia are reasonable means to reduce the use of this scarce resource (40).

### Strengthens of the study

The present study approaches a landmark toward the association of different types between blood transfusion and POD in a large cohort of older patients undergoing TKHA. The general overview of the main highlights of this study is as follows. First, we reviewed all relevant and eligible cases, acquired a few missing pieces of information. Second, the present study demonstrates that the administration of intraoperative allogeneic blood transfusions in older patients increases the risk of developing POD on the first postoperative day. This is not the first study to report an association between intraoperative blood transfusions and POD, but it is the first to identify allogeneic blood transfusion as the strongest predictor of early POD in TKHA. We hope it may provide the foundation for future investigations.

### Study limitations

This study has inherent limitations with potential implications for its interpretation. First, although patient data were recorded perioperatively, they were reviewed retrospectively and were susceptible to the typical weaknesses of retrospective review. The definition of POD using descriptive words in medical chart instead of valid tools such as CAM may overlook the delirium, and the association between different types of blood transfusion and POD needs to be confirmed in further well-designed prospective study in future. Second, factors such as physical therapy, ambulation, daily activities, social support, and inpatient POD precautions during the postoperative period were not recorded, which may also have implications for our results. Third, the study cohort was limited to patients undergoing TKA and THA, who may have been subject to unique perioperative outcomes and pathophysiological mechanisms. Future studies should consider incorporating multicenter data to investigate a more representative group of patients.

## Conclusions

Following elective TJA surgery, POD was observed in 6.6% of patients receiving intraoperative allogeneic blood transfusions and 2.0% of that receiving autologous blood transfusions. Our study suggests an independent association between intraoperative blood transfusions and POD after TJA, with the highest risk associated with allogeneic blood transfusions. However, since this study is retrospective in nature, it is necessary to conduct a well-designed large-scale multicenter randomized controlled trial in the future to further investigate this matter.

## Data Availability

The original contributions presented in the study are included in the article, further inquiries can be directed to the corresponding author/s.
